# Towards pan-genome read alignment to improve variation calling

**DOI:** 10.1186/s12864-018-4465-8

**Published:** 2018-05-09

**Authors:** Daniel Valenzuela, Tuukka Norri, Niko Välimäki, Esa Pitkänen, Veli Mäkinen

**Affiliations:** 1 Department of Computer Science, Helsinki Institute for Information Technology HIIT, University of Helsinki, P.O. Box 68 (Gustaf Hällströmin katu 2b), Helsinki, 00014 Finland; 2 Department of Medical and Clinical Genetics, Genome-Scale Biology Program, University of Helsinki, Helsinki, Finland; 3 European Molecular Biology Laboratory, Genome Biology Unit, Heidelberg, Germany

**Keywords:** Pan-genome reference, Variation calling, Read alignment

## Abstract

**Background:**

Typical human genome differs from the reference genome at 4-5 million sites. This diversity is increasingly catalogued in repositories such as ExAC/gnomAD, consisting of >15,000 whole-genomes and >126,000 exome sequences from different individuals. Despite this enormous diversity, resequencing data workflows are still based on a single human reference genome. Identification and genotyping of genetic variants is typically carried out on short-read data aligned to a single reference, disregarding the underlying variation.

**Results:**

We propose a new unified framework for variant calling with short-read data utilizing a representation of human genetic variation – a pan-genomic reference. We provide a modular pipeline that can be seamlessly incorporated into existing sequencing data analysis workflows. Our tool is open source and available online: https://gitlab.com/dvalenzu/PanVC.

**Conclusions:**

Our experiments show that by replacing a standard human reference with a pan-genomic one we achieve an improvement in single-nucleotide variant calling accuracy and in short indel calling accuracy over the widely adopted Genome Analysis Toolkit (GATK) in difficult genomic regions.

## Background

Accurate identification and genotyping of genetic variation, or variation calling, in high-throughput resequencing data is a crucial phase in modern genetics studies. Read aligners [1–3] have been successful at aligning short reads to a reference genome (e.g. GRCh37). Among the many analyses downstream of read alignment, here we focus on variation calling. Variation calling is the process of characterizing one individual’s genome by finding how it differs from the other individuals of the same species. The standard approach is to obtain a set of reads from the donor and to align them against a single reference genome. The most recent human reference genome, GRCh38, improves on the previous reference version GRCh37 in many respects, including mitochondrial and centromeric sequence quality. Despite containing alternative haplotypes for certain loci, GRCh38 is still largely a haploid consensus reference sequence. Thus, it has been meant to be supplemented by the various databases capturing human genetic variation. Following the alignment of short reads to the reference, multiple tools may be utilized to call variants with respect to the genome (e.g., [4–6]).

However, our current knowledge about the human genome is pan-genomic [7]: after the first human genome was sequenced, the cost of sequencing has decreased dramatically, and today many projects are curating huge genomic databases. These efforts include the 1000 Human Genomes Project [8], UK10K [9], and the Exome Aggregation Consortium and the genome Aggregation Database (ExAC/gnoMAD) [10], the latter consisting of 126,216 exome sequenced and 15,136 whole-genome sequenced individuals. These efforts have already had a significant impact on population and disease genetics. For instance, the pathogenicity of many suspected predisposition variants has been questioned after the discovery of the variants to be relatively frequent in the human population [10]. Supplementing this burgeoning data are the sequencing efforts focusing on phenotypes, for example cancer [11].

In order to align reads to the pan-genome we use pan-genomic indexing [12–20]. That is, instead of having one reference sequence, an entire collection of sequences is indexed, allowing the reads to be mapped against any genome of the reference set or even to some recombination of them.

There is no consensus about how to represent a pan-genome [7]. Previous efforts can roughly be categorized into three classes: one can consider (i) a graph representing a reference and variations from it, (ii) a set of reference sequences, or (iii) a modified reference sequence.

An example of class (i) approach to pan-genomic indexing is to represent the pan-genome as a graph that recognizes all possible variation combinations (population automaton), and then use an extension of the Burrows-Wheeler Transform to support efficient read alignment [16]. Experiments on variation-rich regions of human genome show that the read alignment accuracy is greatly improved over the standard approach [16]. An important caveat of this approach is the indexing phase: the size of the index is exponential in the worst case. Thus, typically it is necessary to drop some variants to achieve a good expected case behavior [16]. Alternatively, one can enumerate all close-by variant combinations and index the resulting variant contexts (i.e. short subpaths in population automaton) in addition to the reference [12, 14, 17, 18]. Yet, in these approaches, the context length needs to be short to avoid exponential blowup.

Class (ii) approaches consider the pan-genome as a set of individual genomic sequences [13, 15, 21]. The Burrows-Wheeler Transform of those sequences is of linear size and the shared content among individuals translates into highly compressed indexes. Lately, there have been proposals to use Lempel-Ziv indexing to obtain an extremely well compressed index that support efficient read alignment [15, 21, 22].

Class (iii) approaches aim at modifying the reference or encoding variants into the reference to improve read alignment accuracy [14, 20].

The scalability of indexed approaches building on the simple class (ii) model of a set of sequences makes them attractive choice as a basis of variation calling. Unfortunately, unlike with class (i) and class (iii) approaches, the literature on them has primary concentrated on the time and space efficiency aspects, neglecting the final goal of enhancing variation calling. This article aims to fill this gap: We propose a model that relies on the class (ii), and we show that by adding little structure to it we can design a flexible pipeline for variation calling that can be seamlessly incorporated into sequencing data analysis workflows.

**Fig. 1:**
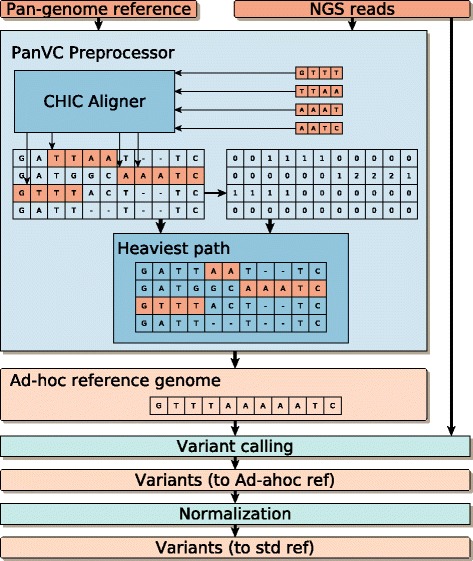
Schematic view of our PanVC workflow for variation calling, including a conceptual example. The pan-genomic reference comprises the sequences GATTATTC, GATGGCAAATC, GTTTACTTC and GATTTTC, represented as a multiple sequence alignment. The set of reads from the donor individual is GTTT, TTAA, AAAT and AATC. CHIC aligner is used to find the best alignment of each read. In the example, all the alignments are exact matches starting in the first base of the third sequence, the third base of the first sequence, the seventh base of the second sequence, and on the eight base of the second sequence. After all the reads are aligned, the score matrix is computed by incrementing the values of each position where a read aligns. With those values, the heaviest path algorithm extracts a recombination that takes those bases with the highest scores. This is the ad hoc genome which is then used as a reference for variant calling using GATK. Finally the variants are normalized so that they are using the standard reference instead of the ad hoc reference

We represent the pan-genome reference as a multiple sequence alignment and we index the underlying set of sequences in order to align the reads to the pan-genome. After aligning all the reads to the pan-genome we perform a read pileup on the multiple sequence alignment of reference genomes. The multiple sequence alignment representation of the pan-genome lets us extract a linear ad hoc reference easily (see “Methods” section). Such a linear ad hoc reference represents a possible recombination of the genomic sequences present in the pan-genome that is closer to the donor than a generic reference sequence. The ad hoc reference is then fed to any standard read alignment and variation detection workflow. Finally, we need to normalize our variants: after the previous step, the variants are expressed using the ad hoc reference instead of the standard one. The normalization step projects the variants back to the standard reference. Our overall scheme to call variants is illustrated in Fig. 1.

## Results

PanVC, our method for variant calling aligns the reads against multiple reference genomes (represented as a multiple sequence alignment) using by default CHIC aligner, a read aligner that specializes in repetitive collections [23]. Using those alignments, it generates an ad hoc reference which is given to GATK workflow instead of the standard reference (See Fig. 1 and “Methods” section). In our experiments, this approach is labeled MSA _*chic*_. As an alternative, we implemented a PanVC version that does not rely on CHIC Aligner, but instead, uses BWA to align against each sequence in the reference. This approach is labelled MSA _*base*_

Additionally, we also compare against the pan-genome reference graph approach [16], which we modified also to output an ad hoc reference (see “Methods” section), so that one can apply the same GATK workflow also for that. This approach is labelled GRAPH.

Finally, as a baseline, we considered GATK workflow [4] that aligns the reads against a reference genome using BWA and analyses the resulting read pileup. This baseline approach is labelled GATK.

### Experimental setup

Our experimental setup consists of a hidden donor genome, out of which a set of sequencing reads is given as input to the variation calling prediction workflows. Our framework PanVC, and also the graph-based approach will use reference set of 20, 50 and 186 genomes. GATK baseline method is limited to use only one reference.

Our experiments focus on variation calling on complex regions with larger indels and/or densely located simpler variants, where significant improvements are still possible. The reason for that is that graph-based pan-genome indexing has been already thoroughly evaluated [16] for mapping accuracy on human genome data. From those results one can infer that on areas with isolated short indels and SNVs, a regular single-reference based indexing approach with a highly engineered alignment algorithm might be already sufficient.

Therefore, we based our experimental setup on the analysis of highly-polymorphic regions of the human genome [24, 25] that was created in a previous study [16]. This test setup consists of variation-rich regions from 93 genotyped Finnish individuals (1000 genomes project, phase 1 data). The 93 diploid genomes gave us a multiple alignment of 186 strains plus the GRCh37 consensus reference.

We chose variation-rich regions that had 10 SNVs within 200 bases or less. The total length of these regions was 2.2 MB. To produce the ground-truth data for our experimental setup, we generated 221559 100 bp single-end reads from each of the Finnish individuals giving an average coverage of 10*x*.

### Evaluation

All evaluated methods output variation calling results that are projected with respect to the standard reference genome. Our hidden donor genome can also be represented as a set of variants with respect to the standard reference genome. This means that we can calculate the standard prediction success measures such as precision and recall. For this, we chose to define the prediction events per base, rather than per variant, to tolerate better invariances of variant locations as have been found to be critical in a recent study [26] (See “Methods” section, “Experimental set-up”).

In addition to precision and recall, we also compute the unit cost edit distance of the true donor and the predicted donor. This is defined as the minimum amount of single base substitutions, insertions, or deletions required to convert the predicted donor into the true donor. Here the sequence content of the true donor is constructed by applying its set of variants to the standard reference and the sequence content of the predicted donor is constructed by applying the predicted variants to the standard reference.

There are good incentives to use this evaluation measure to complement precision and recall: first, it gives a single number reflecting how close the predicted sequence is to the ground truth. Second, the projection from the ad hoc reference to the standard reference may loose information. Third, repeat- and error-aware direct comparison of indel variant predictions is non-trivial and only handled properly on deletions [26].

As our experiments are on human data, where genomes are diploids, the heterozygous variants may overlap, which causes some changes to the evaluation measures above. That is, when applying the variants to the reference, we omit variants that overlap already processed ones, and the result is thus a single sequence consisting of all compatible variants. We follow this approach also when computing the precision and recall measures to make the “per base” prediction events well-defined. The results are illustrated in Tables 1 and 2. Row GATK of Table 1 stands for the GATK workflow. Rows MSA + GATK of Table 1 stand for the multiple sequence alignment -based pan-genome indexing scheme specified in the “Methods” section. Row Graph + GATK of Table 1 is using the graph-based indexing of [16] modified to make it compatible with our workflow. The results are averages over all the donors.

**Table 1:** Edit distance from the predicted donor sequence to the true donor. The average distance between the true donors and the reference is 95193,9

	Pan-genome reference size			
	1	20	50	100
GATK	74695.9	-	-	-
MSA _*base*_ + GATK	-	2885.5	1956.9	1204.7
MSA _*chic*_ + GATK	-	1349.3	1117.4	1099.3
Graph +GATK	-	3230.4	3336.8	2706.9

**Table 2:** Precision and recall of our method MSA _*chic*_ compared to GATK

Measure	GATK	20	50	100
SNV precision	0.992161	0.998585	0.998863	0.998773
SNV recall	0.904897	0.997098	0.998695	0.999072
Indel precision	0.364853	0.996514	0.99731	0.997778
Indel recall	0.0624981	0.982659	0.985723	0.985958

## Discussion

Our results indicate that using pan-genome indexing improves variation calling significantly on highly-polymorphic regions of the human genome: the edit distance between the predicted donor and the true donor is much smaller already when 10 references are used in place of one, and it keeps decreasing when more references are used. When the evaluation metric is precision and recall, the same behavior is observed. In particular, indel calls are improved significantly after the use of pan-genome indexing. Our results reconfirm previous findings about the graph-based approach to pan-genome indexing for specific problems [12, 18]. The approach of tailoring the reference has recently been reported to be beneficial even without using any pan-genomic information; an iterative process to augment a reference and realign has been studied in [19].

A unique feature of our proposal is its genericity. For example, our approach works both on graph representations and on multiple alignment representations of a pan-genome. Earlier studies on pan-genome indexing have mostly focused on read alignments, which are then normalized to the reference to achieve compatibility with the existing variant calling workflows. Instead, here we proposed to globally analyse all read alignments and to produce an ad hoc reference that can be used in place of the standard reference. We keep the projection between the ad hoc reference and the standard reference, so that the variation calling results can always be normalized to the standard reference afterwards.

In addition to variation calling, our methods could be extended to other applications such as to support haplotype analysis in a similar way to a previous study [18]. Namely, one can modify the heaviest path algorithms to produce two predictions. One way to do this is to remove the coverages along the path of the first ad hoc reference and run the heaviest path algorithm again to produce a second ad hoc reference. We leave as future work to make our method fully scalable. We have tested it on multiple alignments of size 1000 times a human chromosome, and with such enormous data sets our analysis pipeline takes weeks to run on a high-performance computer with 1.5 TB of main memory. The current version of our software already contains several engineering solutions to optimize the space usage of intermediate result files and exploit parallelism for maximum speed. Together with our collaborators we are also working on a fully distributed version of the pan-genome analysis pipeline. However, already in its current shape, our software is fully functional in restricted settings, such as calling variants in difficult regions of moderate size. Such feature can be incorporated in a full genome analysis workflow, that processes easy regions using more standard techniques.

## Conclusions

Prior work has focused on graph representations of pan-genomes, usually for specific regions [18]. We show that a multiple sequence alignment can be used as a practical alternative, to keep the structure of a pan-genomic reference.

Our experiments show that by replacing a standard human reference with a pan-genomic one we achieve an improvement in single-nucleotide variant calling accuracy and in short indel calling accuracy over the widely adopted Genome Analysis Toolkit (GATK) in difficult genomic regions.

## Methods

In the following we provide a detailed description of each component of our workflow (Fig. 1). Our scheme is designed to be modular, and to be used in combination with any variation calling workflow.

The first part of our workflow is the generation of the ad hoc reference. This is done by the preprocessor, using as an input the raw reads of the donor as an input and the pan-genome reference.

The second part is to actually call the variants. We don’t provide any details on how to do it because we resort to a variant calling workflow, using our ad hoc reference instead of the standard one. In our experiments, we resort to GATK [4].

Finally, we need to normalize our variants. After the previous step the variants are expressed using the ad hoc reference instead of the standard. The normalization step uses metadata generated from the preprocessor to project the variants back to the standard reference.

### Pan-genome preprocessor

The main role of the pan-genome preprocessor is to extract an ad hoc reference sequence from the pan-genome using the reads from the donor as an input.

#### Pan-genome representation

Following the literature reviewed in the Background section, the existing pan-genome indexing approaches for read alignment could be classified as follows. Some approaches consider the input as a set of sequences, some build a graph or an automata that models the population, and others consider the specific case of a reference sequence plus a set of variations. However, the boundaries between these categories are loose, as a set of sequences could be interpreted as a multiple sequence alignment, which in turn could be turned into a graph. Our scheme can work with different pan-genome representations and indexes provided that it is possible to model recombinations. The multiple sequence alignment and graph representations are versatile enough, but just a collection of sequences is not.

We consider our input pan-genome as a multiple sequence alignment and we store all the positions with a gap. In this way we decouple the problem of book keeping the structure of the pan-genome (in our case, as a multiple sequence alignment) and the problem of indexing the set of underlying sequences.

To transform one representation into the other and to be able to map coordinates we store bitmaps to indicate the positions where the gaps occur. Consider our running example of a multiple alignment



We may encode the positions of the gaps by four bitvectors:



Let these bitvectors be *B*_1_,*B*_2_,*B*_3_, and *B*_4_. We extract the four sequences omitting the gaps, and preprocess the bitvectors for constant time rank and select queries [27–29]: rank_1_(*B*_*k*_,*i*)=*j* tells the number of 1s in *B*_*k*_[1..*i*] and select_1_(*B*_*k*_,*j*)=*i* tells the position of the *j*-th 1 in *B*_*k*_. Then, for *B*_*k*_[*i*]=1, rank_1_(*B*_*k*_,*i*)=*j* maps a character in column *i* of row *k* in the multiple sequence alignment to its position *j* in the *k*-th sequence, and select_1_(*B*_*k*_,*j*)=*i* does the reverse mapping, i.e. the one we need to map a occurrence position of a read to add the sum in the coverage matrix.

These bitvectors with rank and select support take *n*+*o*(*n*) bits of space for a multiple alignment of total size *n* [27–29]. Moreover, since the bitvectors have long runs of 1s (and possibly 0s), they can be compressed efficiently while still supporting fast rank and select queries [30, 31].

#### Pan-genome indexing and read alignment

Now, the problem of indexing the pan-genome is reduced to index a set of sequences.

To demonstrate our overall scheme, we first use a naive approach to index the pan-genome as a baseline: we index each of the underlying sequences individually using BWA [1]. This approach does not offer a scalable pan-genome indexing solution, but it provides a good baseline for the accuracy that one can expect from a true pan-genome indexing solution to provide. In our experiments, this approach is labeled MSA _*base*_.

For a scalable solution that can manage large and highly repetitive set of references we resort to CHIC aligner [23], which combines Lempel-Ziv compression to remove the redundancy with a Burrows-Wheeler index to align the reads. In our experiments, this approach is labeled MSA _*chic*_.

#### Heaviest path extraction

After aligning all the reads to the multiple sequence alignment, we extract a recombined (virtual) genome favoring the positions where most reads were aligned. To do so we propose a generic approach to extract such a heaviest path on a multiple sequence alignment. We define a score matrix *S* that has the same dimensions as the multiple sequence alignment representation of the pan-genome. All the values of the score matrix are initially set to 0.

We use CHIC aligner to find the best alignment for each donor’s read. Then we process the output as follows. For each alignment of length *m* that starts at position *j* in the genome *i* of the pan-genome, we increment the scores in *S*[*i*][*j*],*S*[*i*][*j*+1]…*S*[*i*][*j*+*m*−1] (adjusting the indexes using the bit-vector representations considered in the previous subsection). When all the reads have been processed we have recorded in *S* that the areas with highest scores are those where more reads were aligned. An example of this is shown in Fig. 1.

Then we construct the ad hoc reference as follows: we traverse the score matrix column wise, and for each column we look for the element with the highest score. Then, we take the nucleotide that is in the same position in the multiple sequence alignment and append it to the ad hoc reference. This procedure can be interpreted as a heaviest path in a graph: each cell (*i,j*) of the matrix represents a node, and for each node (*i,j*) there are *N* outgoing edges to nodes (*i*+1,*k*), *k*∈{1,…,*N*}. We add an extra node *A* with *N* outgoing edges to the nodes (1,*k*), and another node *B* with *N* ingoing edges from nodes (*L,k*). Then the ad hoc reference is the sequence spelled by the heaviest path from *A* to *B*. The underlying idea of this procedure is to model structural recombinations among the indexed sequences.

A valid concern is that the resulting path might contain too many alternations between sequences in order to maximize the weight.

To address this issue there is a simple dynamic programming solution to extract the heaviest path, constrained to have a limited number of jumps between sequences: Consider a table *V*[1…*L*][1…*N*][0…*Z*] initially set to 0. The values *V*[*i,j,k*] correspond to the weight of the heaviest path up to character *i*, choosing the last character from sequence *j*, that has made exactly *k* changes of sequences so far. The recursion for the general case (*k*>0, *i*>1) is as follows: $\phantom {\dot {i}\!} V[i,j,k] = S[i,j] + max \{ V[i-1,j,k], max_{j' \neq j} V[i-1,j',k-1] \}$, and the base case for *k*=0, *i*>1 is: *V*[*i,j*,0]=*S*[*i,j*]+*V*[*i*−1,*j*], and for *k*=0, *i*=1: *V*[1,*k*,0]=*S*_1,*j*_.

Once the table is fully computed, the weight of the heaviest path with at most *k*^∗^ changes is given by *max*_*j*_{*V*[*L,j,k*^∗^]}. To reconstruct the path we need to traceback the solution.

However, in our experiments we noticed that the unconstrained version that just selects a maximum weight path without additional constraints performs better than the constrained version, and so we use the former by default in our pipeline.

**Fig. 2:**
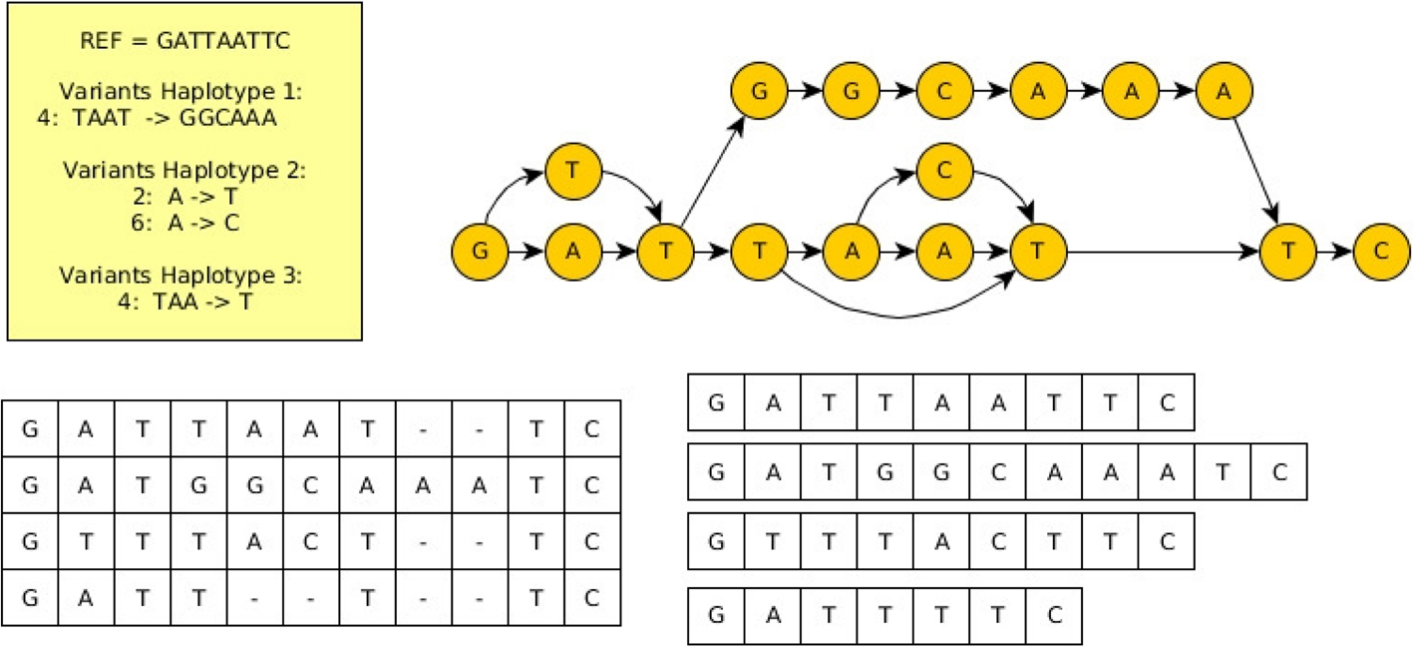
Four different representations of a pan-genome that corresponds to the same set of individuals. Top left: a reference sequence plus a set of variants to specify the other individuals. Top right: a (directed acyclic) graph representation. Bottom left: a multiple sequence alignment representation, Bottom right: a set of sequences representations

It is worth noting that as opposed to a graph representation of the pan-genome where the possible recombinations are limited to be those pre-existing in the pan-genome, our multiple sequence alignment representation can also generate novel recombinations by switching sequences in the middle of a pre-existing variant. This happens in our example in Fig. 1, where the ad hoc reference could not be predicted using the graph representation of the same pan-genome shown in Fig. 2.

### Variant calling

Variant calling can be in itself a complex workflow, and it might be tailored for specific type of variants (SNVs, Structural Variants), etc. We aim for a modular and flexible workflow, so any workflow can be plugged in it. The only difference is that we will feed it the ad hoc reference instead of the standard one.

In our experiments, we used GATK [4] version 3.3, following the Best Practices: first we aligned the reads to the reference using BWA, and next we used Picard to sort the reads and remove duplicates. Then we performed indel realignment using GATK RealignerTargetCreator and IndelRealigner, and finally we called variants using GATK HaplotypeCaller using parameters genotyping mode = DISCOVERY, standemit conf = 10 and standcall conf = 30.

### Normalizer

Finally we need to normalize our set of variants. To do so we apply the variants to the ad hoc reference, so that we obtain an alignment between the ad hoc reference and the predicted sequence. The metadata generated in the preprocessor stage – while extracting the heaviest path – includes an alignment between the standard reference and the ad hoc reference. Using those, we can run a linear-time algorithm to obtain an alignment between the standard reference and the predicted sequence. From this alignment, we can generate a vcf file that expresses the predicted sequence as a set of variants from the standard reference.

### Experimental set-up

#### Evaluation metric

We separate the single nucleotide variant (SNV) calls from indel calls as the results differ clearly for these two subclasses. A true positive (TP) SNV call is a SNV in the true donor and in the predicted donor. A false positive (FP) SNV call is not a SNV in the true donor but is a SNV in the predicted donor. A false negative (FN) SNV call is a SNV in the true donor but is not a SNV in the predicted donor. A true positive (TP) indel call is either an inserted base in the true donor with an identical inserted base in the predicted donor, or a deleted base in both the true and predicted donor. A false positive (FP) indel call is neither inserted nor deleted base in the true donor but is either inserted or deleted base in the predicted donor. A false negative (FN) indel call is an inserted or deleted base in the true donor but is neither inserted nor deleted base in the predicted donor. We report precision=TP/(TP+FP) and recall=TP/(TP+FN).

#### Modification to graph representation of pan-genome

In our approach we have used a multiple sequence alignment to represent the pan-genomic reference, but it is relatively easy to to use a graph representation [16] instead. A graph representation of a pan-genome usually use a vertex-labeled directed acyclic graph (labeled DAG), and reads are aligned to the paths of this labeled DAG. After all the reads have been aligned to the pan-genome, instead of our score matrix, we can store for each vertex the number of read alignments spanning it. Then the heaviest path can be easily computed using dynamic programming in a topological ordering of the graph: the weight of the heaviest path *h*(*v*) to a vertex *v* is $\max _{v' \in N^{-}(v)} h(v')+w(v)$, where *w*(*v*) is the weight of a vertex and *N*^−^(*v*) is the set of vertices connected with a in-coming arc to *v*.

The difference to the multiple alignment heaviest path is that the number of recombinations cannot be limited when using the graph representation.

Another part that is different is the normalizer module to map the variants predicted from the ad hoc reference to the standard reference. For this, the original proposal in [16] already records the path spelling the standard reference, so while extracting the heaviest path one can detect the intersection to the standard reference path and store the corresponding projection as an alignment. Thus, one can use the same evaluation metrics as in the case of multiple sequence alignment -based variation calling.

### Data availability

The datasets generated during and/or analysed during the current study are available from the corresponding author on reasonable request; most of the data and scripts to replicate the experiments, as well as a pre-built pan-genome index for the 1000 Human Genomes project data, are available online: https://www.cs.helsinki.fi/gsa/panVC

### Code availability

Our tools are open source and available online: https://gitlab.com/dvalenzu/PanVC

## Abbreviations

DAG: Directed acyclic graph
FN: False negative
FP: False positive
GATK: Genome analysis toolkit
MSA: Multiple sequence alignment
SNV: Single nucleotide variant
TN: True negative
TP: True positive
